# Quantifying Lateral Pulsation in Retinal Vessels Within the Optic Disc

**DOI:** 10.1167/tvst.15.7.20

**Published:** 2026-07-14

**Authors:** Aleksandar Vukmirovic, William H. Morgan, Danail Obreschkow, Anmar Abdul-Rahman, Dao-Yi Yu, Andrew Mehnert

**Affiliations:** 1 Lions Eye Institute, Centre for Ophthalmology and Visual Science, University of Western Australia, Crawley (Perth), Western Australia; 2 International Space Centre, Crawley, Western Australia; 3 International Centre for Radio Astronomy Research (ICRAR), M468, University of Western Australia, Crawley (Perth), Western Australia; 4 Department of Ophthalmology, Counties Manukau DHB, Auckland, New Zealand

**Keywords:** lateral pulsation, retinal vessels, optic disc, non-rigid registration, pulse waves

## Abstract

**Purpose:**

The retina provides a noninvasive window for imaging vascular pulsation in the optic disc. Pulsation characteristics reflect vascular stiffness, hemodynamics, and ocular pressure dynamics, useful in assessing ocular and systemic cardiovascular health. Whereas axial pulsation can be quantified using modified photoplethysmography (PPG), no reliable method exists for quantifying lateral pulsation. Here, we present a method.

**Methods:**

Video of the optic disc is acquired over multiple cardiac cycles. Frames are globally aligned and non-rigidly warped to a common space, then averaged to form a template image from which the vessel of interest is segmented. For each frame, the inverse warp is used to compute sub-pixel vessel wall displacements relative to the template. The method and PPG were applied to a vertically oriented vein in one eye from three healthy subjects. Harmonic regression models were used to compare the vessel wall displacement and diameter pulse waves, with the axial pulse wave from PPG.

**Results:**

Where the models fitted well (pseudo-*R*^2^ ≥ 75%), the lateral pulse waves were either in phase with the axial pulse wave or showed a small phase delay, with the median phase difference ranging from 4.4% to 9.2% of the cardiac cycle (all *P* ≤ 0.04). The largest difference between left- and right-wall displacement magnitudes (asymmetric displacement) for each subject were 7.6, 25.2, and 6.33 µm respectively.

**Conclusions:**

The results demonstrate the efficacy of the method for quantifying lateral pulsation within the optic disc.

**Translational Relevance:**

The proposed method demonstrates proof-of-concept feasibility for quantifying lateral pulsation within the optic disc and lays the groundwork for future biomarker investigation.

## Introduction

The retina offers a unique noninvasive window through which microvasculature pulsation, reflecting underlying physiological and pathological processes, can be imaged and quantified.^1^^,^^2^ Changes in retinal vascular pulsation and vessel geometry are implicated in a range of diseases such as diabetic retinopathy and cardiovascular disease.^3^^–^^6^ Both axial and lateral pulsations can be observed (axial here refers to the optical axis of the imaging system). Axial pulsation is traditionally characterized by changes in image intensity through techniques such as modified photoplethysmography, whereas lateral pulsation presents as in-plane displacements of the vessel walls. Interestingly, these lateral displacements may be either symmetric or asymmetric. Existing methods for quantifying retinal vessel lateral pulsation do not quantify asymmetric pulsation. Moreover, there is currently no reliable method for quantifying lateral pulsation within the optic disc, where vascular pulsation is most pronounced due to the complex interplay among intraocular pressure, cerebrospinal fluid pressure, and intravascular pressure, modulated by vessel wall and local tissue mechanics.

To date, dynamic changes in retinal vessel diameters have primarily been characterized using three approaches. The first approach directly estimates lateral diameter changes of retinal vessels. One example of this approach is the commercially available dynamic vessel analyzer (DVA).^7^ It is an ophthalmic instrument consisting of a fundus camera equipped with a CCD camera, which uses image processing to measure retinal vessel diameters across time and space.^7^ It does so by analyzing the intensity profiles across the vessel cross-sections to detect the vessel edges and calculating the distance between them.^7^ The DVA has also been adapted for analyzing vessel diameter measurements in scanning laser ophthalmoscope (SLO) images.^8^ Another example is the SLOctolyzer designed to measure vessel diameters in infrared SLO images.^9^ Like the DVA, it detects vessel edges and measures the distance between them. The robustness of this intensity profile-based approach depends on the reliability with which vessel edges can be detected. For example, it has been reported that DVA is unable to reliably delineate the edges of retinal vessels within the optic disc in fundus ophthalmoscope images.^10^

The second approach is that of Spahr et al., which uses a phase sensitive full field swept source optical coherence tomography (PhS-FF-SS-OCT) technique to estimate axial retinal motion based on temporal changes in the phase of the OCT signal.^11^ The phase shifts within the signal enable this method to measure axial retinal tissue displacement with nanometer level precision.^11^ However, PhS-FF-SS-OCT has several limitations. First, it requires highly specialized hardware, including high-speed cameras and swept laser sources that are costly and not widely available. Second, phase stability is highly sensitive to timing mismatches and sweep to sweep variations in the laser source which can introduce significant phase errors and require complex signal correction strategies.^11^^,^^12^ Last, the lateral resolution of the vessel is decreased by the need for spatial averaging to improve the signal to noise ratio and as such reduces the ability of this technique to resolve localized vessel wall dynamics.^11^

The final approach uses a modified video photoplethysmography (PPG) technique that induces venous pulsation through externally applied pressure on the eye, enabling quantification of intensity changes within retinal vessels. The intensity can be related to the blood column thickness via the Beer Lambert law, providing an indirect measurement of axial vessel diameter.^13^^–^^15^

Here, we present a feasibility study for a novel approach for quantifying sub-pixel lateral vessel wall displacements along retinal vessel walls within the optic disc using videos of the optic disc acquired at 25 frames per second for one or more cardiac cycles. Venous pulsation is induced through externally applied pressure on the eye to enable quantification of vessel diameter changes along the vessel. Rather than explicitly locating vessel edges in each video frame, the method is based on group-wise non-rigid registration. Each image frame is warped to a common coordinate space, the registered images averaged to create a template image, the vessel of interest is segmented to generate a vessel mask, and the sub-pixel vessel wall displacements with respect to this mask are computed using the estimated displacement fields. The method is also suitable for characterizing lateral pulsation within the optic disc.

## Materials and Methods

In this section, we present our method for measuring sub-pixel lateral vessel wall displacements along retinal vessels over time from a video of the optic disc. We also describe an empirical evaluation of the method against PPG using data acquired from three eyes of three subjects.

### Method for Measuring Lateral Retinal Vessel Wall Pulsation Using Videos of the Optic Disc

**Figure 1.**
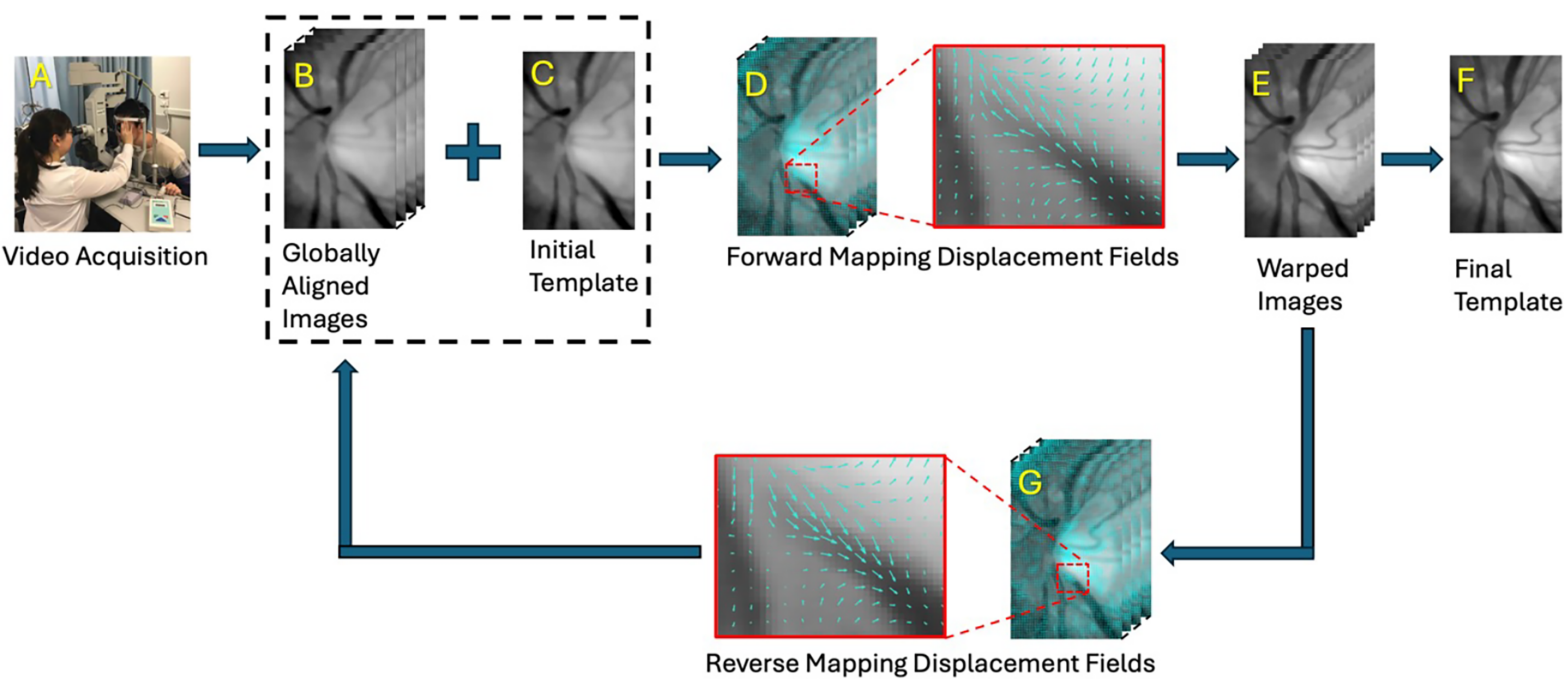
Stage 1: Warping the *green* channel video frames to a common reference space. (**A**) Video frames are captured over three cardiac cycles. (**B**) The stack of *green* channel video frames is globally aligned (affine registration) to the sharpest frame. (**C**) The mean of the frames is used as the initial template for groupwise non-rigid registration. (**D**) Resulting stack of 2D displacement fields (shown as overlaid quiver plots with *cyan* vectors) describing the forward mapping of each frame to the common coordinate space. (**E**) Stack of warped frames. (**F**) The final template is the mean of the warped frames. (**G**) Stack of 2D displacement fields describing the reverse mapping of each warped frame in **E** back to the corresponding globally aligned frame in **B** obtained using pairwise non-rigid registration.

**Figure 2.**
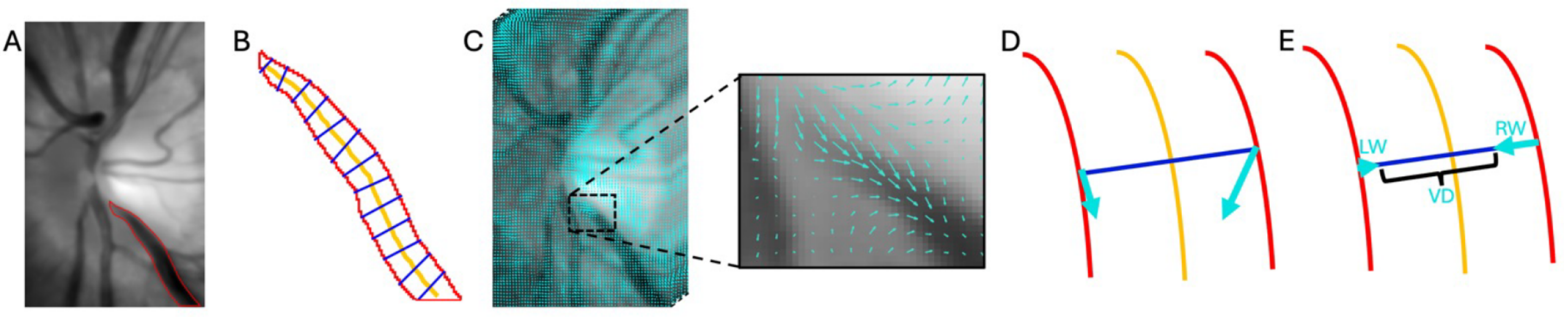
Stage 2: Schematic showing how the vessel wall displacements and vessel diameter changes are calculated. (**A**) The final template obtained using groupwise non-rigid registration in stage 1, with an inferior retinal vein outlined in *red*. (**B**) The vessel outline including the centerline in *orange* extracted from the vessel mask, and orthogonal crosslines in blue along the centerline (only shown for every 10th pixel). (**C**) Lateral displacement fields in the x and y plane from stage 1 for reverse mapping. (**D**) Expanded view of **B**, showing one of the blue crosslines overlaid with the displacement vectors for the endpoints (vessel walls). (**E**) Projections of the displacement vectors along the crossline. The left wall (LW) and right wall (RW) displacements are defined to be the change in position away from the vessel centerline, that is, positive values represent displacement away from the centerline and negative toward. The vessel diameter (VD) is defined to be the sum of the LW and RW displacements, and the crossline length which measures the change in vessel diameter with respect to the template space.

The method comprises two stages. In the first stage (see Fig. 1), the green channel frames are warped to a common reference space with a shared coordinate system. The warping applied to each frame is described by a forward displacement field. The average of these warped frames is taken to be the template image. The reverse displacement fields, mapping each warped image back to the original green channel image, are also determined. In the second stage (see Fig. 2), the template image and the set of reverse displacement fields are used to calculate vessel wall displacements and vessel diameter changes relative to the template image.

#### Stage 1. Warping the Green Channel PPG Frames to a Common Reference Space

The input to stage 1 is a video (temporal stack of image frames) of the retina over three cardiac cycles acquired using the PPG system (see Fig. 1A) we have previously described.^15^^–^^17^ Briefly, the subject is seated at a slit lamp (Carl Zeiss, Germany) with a pulse oximeter attached to the right index finger. An ophthalmodynamometer is used during video acquisition, and the audible oximeter “beep” is recorded simultaneously. Videos are recorded at 25 frames per second using a Canon 5D mark III (Canon Corp, Japan) and typically contain 65 to 75 frames. Each frame is a 24-bit RGB image with a resolution of 1920 × 1080 pixels and pixel aspect ratio of 1.^15^^–^^17^ The frames are cropped to the bounds of the optic disc, approximately 200 *×* 300 pixels, corresponding to an estimated pixel size for the population average eye of 7.6 µm. The pixel size is calibrated based on the height of the optic disc in mm. This is measured using a confocal infrared reflectance image of the same eye's optic disc, captured with the SLO integrated into the Spectralis OCT system.^18^

Next the green channel frames are extracted.^15^^–^^17^ A Laplacian of Gaussian filter is then applied to each image frame and the standard deviation of the pixel intensities in each filtered frame are calculated.^2^ The sharpest frame is then identified as the frame with the largest standard deviation.^2^ Blurry frames with a sharpness value <75% are replaced with the nearest non-blurry frame.^2^ The frames are then globally aligned using a hierarchical registration process (translation-only alignment, followed by rigid registration, and then a full affine transformation including shear) as we have previously described (see Fig. 1B).^15^^–^^17^ Finally, the globally aligned frames are manually reviewed to ensure registration was successful.^15^^–^^17^

Groupwise non-rigid registration is then performed (see pseudocode in the Supplementary Material), finding a transformation (displacement field) for each frame so that they all align to a common reference space with a shared coordinate system. Groupwise registration minimizes bias by aligning multiple frames simultaneously rather than warping them to a single reference frame.^19^ An initial template image is generated by computing the mean of the globally aligned frames (see Fig. 1C). Each frame is then deformably registered or warped to this template. The template is then updated to be the average of the warped frames, and this process is repeated.^19^ Iterations continue until convergence, that is, when the change in the cost function between successive iterations falls below a predefined threshold value. The cost function evaluates groupwise differences in image intensities across the registered image set and incorporates a regularization term that penalizes non-smooth deformation fields.^20^ This iterative process ensures that the warped frames (see Fig. 1E) have achieved an optimal groupwise alignment. The groupwise aligned frames are then manually reviewed to ensure registration was successful. The final template image is the average of the final warped frames (see Fig. 1F). However, MATLAB's “imreggroupwise” does not estimate the inverse displacement field (warping the solution back to the original frame). Moreover, the inverse displacement field cannot be obtained from the forward displacement field due to the inherent non-symmetric nature of the transformations estimated by “imreggroupwise”.^20^^,^^21^ To address this, we perform pairwise registration between the groupwise aligned frames and the original frames to explicitly estimate the inverse displacement field for each frame (Fig. 1G). This allows the displacement fields to be expressed in the native coordinate system of each image, ensuring that the extracted fields accurately represent local vessel motion.

#### Stage 2: Calculation of Vessel Wall Displacements

The input to stage 2 is the final template image and the stack of displacement fields describing the reverse mapping from stage 1. The vessel of interest within the optic disc is manually delineated in the final template image (see Fig. 2A). A one-pixel thick vessel centerline (medial axis) is computed from the binary mask of the vessel. Orthogonal cross lines are then generated for each pixel along the centerline spanning the diameter of the vessel of interest (see Fig. 2B). The coordinates of the crossline endpoints on either side of the centerline are extracted. These coordinates are used to look up the corresponding displacement vectors in each reverse displacement field (one field for each warped frame mapped back to the original set of frames; see Fig. 2C). The set of displacement vectors for each edge coordinate (see Fig. 2D) describe the displacement of that edge relative to the template over time. The displacement vectors are then projected along the normal to the template centerline (see Fig. 2E) at every pixel along the centerline of the vessel of interest for every frame.

#### Implementation

The method was implemented using custom scripts written in FIJI,^22^ R^23^ (version 4.2.2), and MATLAB (version R2023b). The MATLAB functions imreggroupwise and imregdeform were used for groupwise and pairwise registration, respectively.^21^ Both use the total variation regularization to perform deformable registration.^20^

### Empirical Evaluation

The proposed method was applied to 3 healthy young subjects aged from 26 to 34 years old. For each subject, the vessel exhibiting the largest pulsation amplitude within the optic disc was selected. In all three cases, the selected vessels happened to be vertically oriented inferior veins. For one of the cases, a vertically oriented superior vein was additionally selected. The PPG method was also applied to obtain axial vessel diameter measurements. The use of human subjects for the video measurements was approved by Belberry Human Research Ethics Committee (permit number 2015-11-756-A-2), in accordance with the Declaration of Helsinki and in compliance with National Health and Medical Research Council guidelines for clinical trials. All measurements were performed according to relevant guidelines and regulations, and informed consent was obtained from the subject. All measurements were obtained using standard clinical protocols for equipment cleaning and best practice following Lions Eye Institute guidelines. The subject had a prior SLO scan of the optic disc (Heidelberg Engineering, Heidelberg, Germany).

A harmonic regression model, which we have detailed in our previous work,^24^^–^^26^ was fitted using generalized least squares separately to four sets of measurements: left wall (LW) lateral displacements, right wall (RW) lateral displacements, lateral vessel diameter (VD; all in µm), and the negative logarithm of the green color channel pixel intensity as a proxy for the axial VD (in arbitrary units [AU]). The model has the form:

(1) $$\begin{array}{c} y\left(t\right)=f_{p}\left(t\right)+f_{\mathrm{np}}\left(t\right)+\varepsilon_{t} \end{array}$$

where *y*(*t*) is the displacement or diameter measurement in the video frame at time *t*, *f*_p_ is the periodic component, and *f*_np_ is the non-periodic component and is modeled as a linear spline with breakpoints at the start and end of each cardiac cycle. This component captures inter-cycle lighting variations by assuming a piecewise linear intensity trend across cardiac cycles. Lastly, ε_*t*_ is a first-order autoregressive error component.^24^ The time *t* is the fraction of the cardiac cycle rather than time in seconds. The periodic component is modeled as the Fourier series expansion, in sine-cosine form, up to the second harmonic, see below:

(2) $$\begin{array}{c} f_{p}\left(t\right)=a_{0}+\sum_{n=1}^{2}a_{n}\cos\left(2\pi nt\right)+b_{n}\sin\left(2\pi nt\right) \end{array}$$

where *a_i_* and *b_i_* are real coefficients, and *t* represents time as a fraction of the cardiac cycle rather than seconds (the sine-cosine expansion is amenable to fitting using generalised least squares).

For each fitted model, the minimum time to trough (MTT) in cardiac cycle time (i.e. proportion of the cardiac cycle) was computed as well as the pulse wave amplitude (PWA). The former is the cardiac cycle time to the first trough in the plot of the fitted model. The latter is the difference between the maximum and minimum values of the periodic component of the fitted model.^15^ The MTT for each lateral pulse wave was subtracted from the MTT of the axial pulse wave to determine phase difference in cardiac cycle time. For each vein, the largest difference between the magnitudes of the LW and RW displacements was used to identify the location along the vessel with the largest asymmetric vessel wall displacement.

In addition, a curvature-PWA analysis was conducted in one subject (subject A) to demonstrate the potential of this method to study the relationship between pulsation amplitude and vessel curvature. The ability to study PWA in relation to vessel geometry may be useful given that retinal vessel geometry is known to be associated with disease such as diabetic retinopathy.^4^ Whether this analysis is useful or generalizes is an open question. Smoothed splines were fitted separately using R software (spar = 0.7) to the LW and RW of the selected vein in the template image. This permitted the curvature to be calculated at each point along a wall based on the first and second derivatives of the spline.^27^

#### Statistical Analysis

All statistical analyses were performed in R. The package nlme^28^ was used to fit the harmonic regression models using generalized least squares. The package piecewiseSEM was used to compute pseudo-*R*^2^ for the fitted models, which is a measure of goodness-of-fit analogous to *R*^2^ used in ordinary least squares.^29^ Pseudo-*R*^2^ is based on the squared correlation between observed and fitted values. The package ggplot2 was used to plot axial and lateral displacements with their respective fitted harmonic regression model.^30^ The stats package was used to fit the smoothed splines.^31^^–^^33^

Phase differences in cardiac cycle time were summarized using median and interquartile ranges (IQRs) along each vessel from the optic disc center toward the boundary where the regression models fitted well (pseudo-*R*^2^ ≥ 75%). For each subject and lateral pulsation measure (LW, RW, and VD), Wilcoxon signed-rank tests were used to assess whether the estimated pulse waves were in phase with the axial pulse wave. Phase differences were evaluated only where pseudo-*R*^2^ ≥ 75% across all four models.

PWAs were summarized for subject A using median and IQR along the vessel from the optic disc center to the boundary and within the visibly pulsating segment length.

## Results

**Figure 3.**
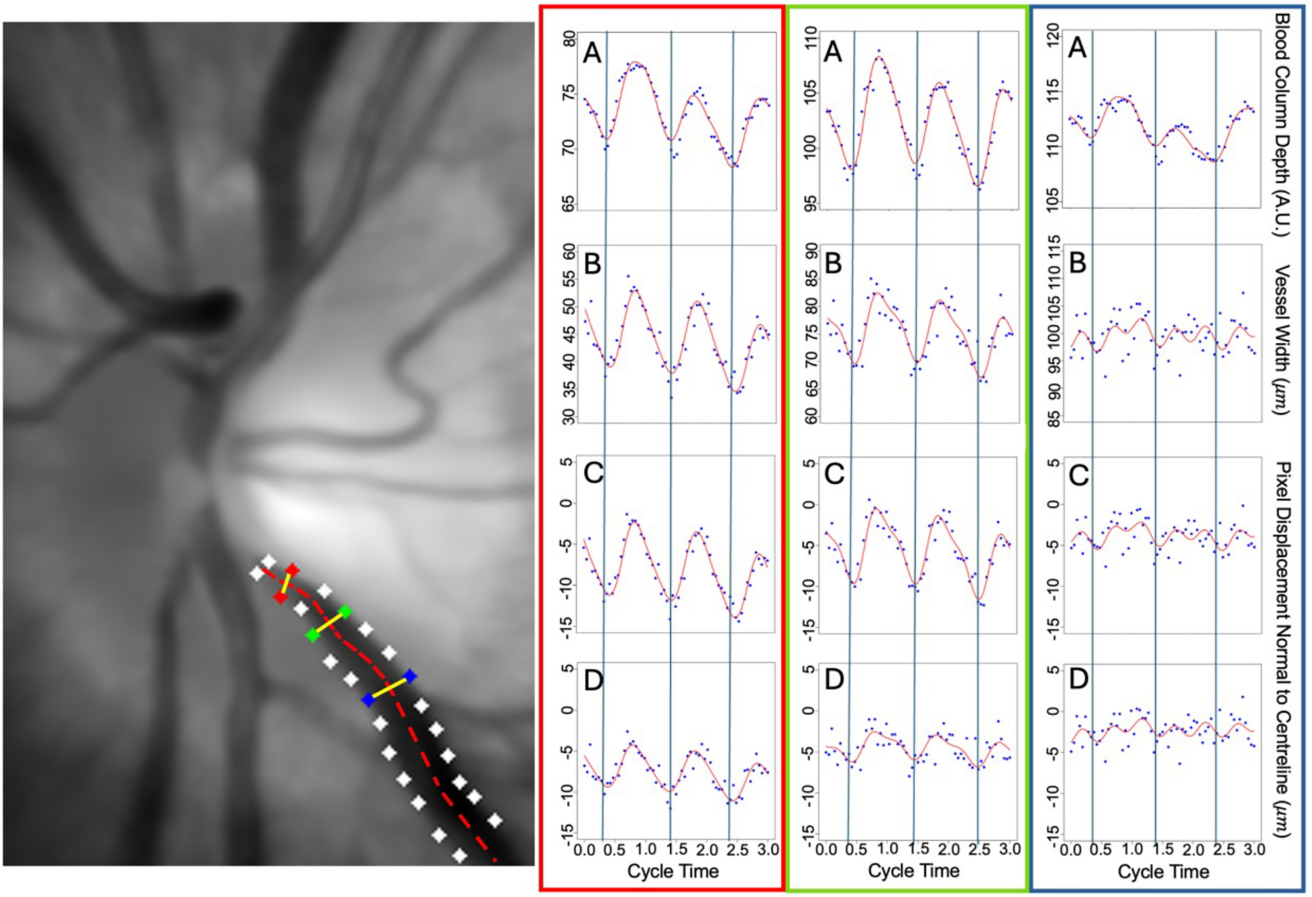
Harmonic regression model fits for a vertically oriented inferior vein of subject A over three cardiac cycles. Every 10th edge pixel is marked with a *white square*. Fits are shown at three *yellow* crosslines located at the 10th, 30th, and 60th pixels along the vein centerline (*red dashed line*) shown in *red*, *green*, and *blue*, respectively. *Blue* points are the raw measurements, and the *red* curve is the fitted model. (**A**) Axial vessel diameter changes (AU). (**B**) Lateral vessel diameter (VD) changes (µm). (**C**) Right wall (RW) displacements (µm). (**D**) Left wall displacements (µm).

Plots of the fitted harmonic regression models for a vertically oriented inferior vein of subject A are presented in Figure 3. Plots of the fitted harmonic regression models for vertically oriented inferior veins of subjects B and C and a vertically oriented superior vein of subject A are presented in Supplementary Figures SA1, SA2, and SA3. Additionally for subject A, we plotted phase versus distance for the left and right edge displacements (Supplementary Figs. SA4 and SA5). These show a progressive phase delay for both vessel edges, yielding estimated pulse wave velocities of 21.14 mm/s and 23.38 mm/s for the left and right edges, respectively.

Table 1 summarizes the pseudo-*R*^2^ values for the length of the vein to the optic disc boundary and within the visibly pulsating segment length for subject A. The harmonic regression model explained more than 75% of the total variance between the observed data and the fitted model across all 4 measurements in the visibly pulsating segment of the vein. Tables summarizing pseudo-*R*^2^ values for subjects B and C are presented in Supplementary Tables SA1 and SA2.

**Table 1.** Summary of Pseudo-*R*^2^ Values for the Regression Models Fitted to the Lateral and Axial Displacement/Diameter Measurements Along the Selected Vessel for Subject A

Measurement	Entire Length of the Vein^a^	Visibly Pulsating Segment^b^
Right wall (RW) displacement	0.46 (0.66)	0.91 (0.01)
Left wall (LW) displacement	0.26 (0.35)	0.78 (0.17)
Lateral vessel diameter (VD)	0.34 (0.61)	0.87 (0.04)
Axial diameter	0.82 (0.15)	0.93 (0.03)

Reported as median (IQR).

a At 579 µm.

b At 181 µm.

Table 2 summarizes the phase differences in cardiac cycle time along each vessel from the optic disc center toward the boundary where the regression models fitted well (pseudo-*R*^2^ ≥ 75%) for all three subjects.

**Table 2.** Summary of Phase Differences (Expressed as a Proportion of Cardiac Cycle Time) Between the Lateral Pulse Waves and Axial Pulse Wave Along the Selected Vein From the Optic Disc Center Toward the Boundary Where all Four Regression Models Fitted Well (Pseudo-*R*^2^ ≥ 75%)

Subject	Right Wall (RW)	Left Wall (LW)	Lateral Vessel Diameter (VD)
A	0.047 (0.00); *P* < 0.001	0.047 (0.00); *P* < 0.001	0.047 (0.00); *P* < 0.001
B	0.000 (0.00); *P* = 0.04	0.092 (0.04); *P* = 0.002	0.000 (0.00); *P* = NA (all paired differences = 0)
C	0.044 (0.04); *P* < 0.001	0.044 (0.04); *P* < 0.001	0.044 (0.04); *P* < 0.001

Values are reported as median (IQR).

*P* values are derived from Wilcoxon signed-rank tests and have not been adjusted for multiple comparisons.

**Figure 4.**
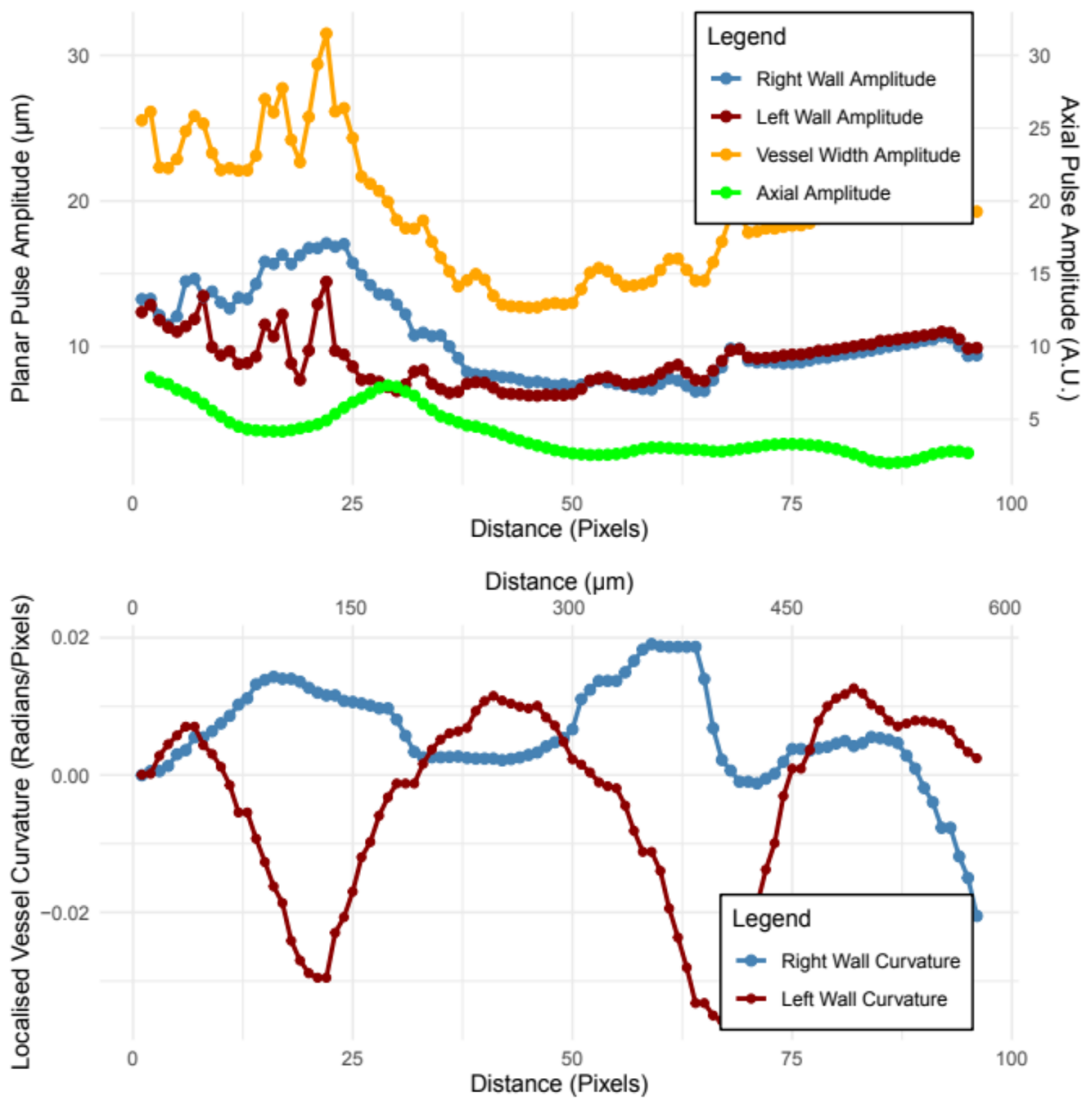
Comparison of the left wall (LW), right wall (RW), and lateral vessel diameter (VD) pulse wave amplitudes (PWAs) with vessel wall curvature along the selected vessel for subject A. The distance is measured along the vessel centerline toward the optic disc boundary.

The largest asymmetric vessel wall displacement, corresponding to the largest difference between the magnitudes of the left wall and right wall displacements, were 7.6 µm for subject A, 25.2 µm for subject B, and 6.33 µm for subject C. Table 3 summarizes the PWAs for subject A along the selected vessel from the optic disc center to the boundary within the visibly pulsating segment length. Figure 4 presents plots of the PWAs for the LW, RW, and lateral VD pulse, and vessel wall local curvatures along the centerline of the selected vein for subject A. The maximum PWA was greater for the right wall than the left wall.

**Table 3.** Summary of the Pulse Wave Amplitudes (PWAs) Along the Entire Length of the Vein and Within the Visibly Pulsating Segment of Subject A

Measurement	Entire Length of the Vein^a^	Visibly Pulsating Segment^b^
Right wall	9.79 µm (IQR 5.20)	14.26 µm (IQR 2.54)
Left wall	9.20 µm (IQR 2.55)	9.72 µm (IQR 2.92)
Vessel diameter	18.93 µm (IQR 6.92)	23.74 µm (IQR 3.87)
Axial diameter	3.28 AU (IQR 2.09)	5.59 AU (IQR 2.30)

Reported as median and IQR.

a At 579 µm.

b At 181 µm.

The maximum PWA among the LW, RW, and lateral VD measurements, was that for the lateral VD occurring at 22 pixels along the vessel or 133 µm from the origin (point closest to the optic disc center). The maximum axial PWA after the origin of the vessel was at 29 pixels along the vessel or 175 µm from the origin. The most positive local curvature (bending away from the centerline) and the most negative local curvature (bending toward the centerline) along the left vessel wall was 0.013 Radians/pixel and –0.036 Radians/pixel respectively. For the right vessel, they were 0.019 Radians/pixel and –0.021 Radians/pixel.

## Discussion

Our method enables the measurement of sub-pixel lateral vessel wall displacements along the length of a vessel within the optic disc from videos of the retina. This permits quantitative characterization of the pulse wave in the lateral plane based on changes in vessel diameter at each point along the vessel centerline, as well as changes in vessel wall displacements on either side of the centerline. The latter makes it possible to characterize asymmetric lateral pulsation.

We compared this new method against the published modified video photoplethysmography technique (which measures changes in axial diameter from optical density changes) in a selected vertically oriented vein within the optic disc in three eyes from three healthy young subjects. Separate harmonic regression models were fitted to the lateral and axial measurements along the vessel. The lateral pulse waves were either in phase with or had a small delay relative to the axial pulse wave (see Table 2). This occurred along the vessel segment closest to the optic disc center, with progressively poorer fits toward the optic disc boundary, where the measured displacements were less pronounced (see Figure 3; Supplementary Figs. S3A1, S3A2, S3A3, Table 1; Supplementary Tables SA1, SA2). Notably, for each of the lateral displacement/diameter measures and axial diameter, the model fits within the visibly pulsating vein segment for all three subjects were all very strong (Table 1; Supplementary Tables SA1, SA2).

An advantage of the new method is that it can quantify lateral vessel wall displacement on either side of the vessel, permitting assessment of asymmetric vessel wall displacement. Our results showed that for all three subjects, there was asymmetric displacement with the RW experiencing greater deformation than the LW. These results suggest that the venous collapse within the optic disc is not uniform but instead reflects varying mechanical properties or external constraints acting asymmetrically on the venous wall.

Asymmetric collapse of the vein within the optic disc is not well understood but while still speculation there are potential explanations for this behavior. Outside of the optic disc boundary, retinal veins lie relatively loosely on the retinal surface.^15^ When retinal veins enter the optic disc and exit into the lamina cribrosa they become firmly tethered to the laminar collagen and their surrounding connective tissue.^15^ Potentially, the tethering of veins may be greater on one side of the venous wall than the other leading to asymmetric collapse. Previous studies have also demonstrated associations among retinal vessel geometry, including tortuosity, and disease states such as diabetic retinopathy.^4^^–^^6^ Furthermore, the geometry of the vessel itself may possibly contribute to asymmetric vessel collapse.^34^ In Figure 4, we see that the segment of the left vessel wall with the most negative curvature near the optic disc center coincides with the point of maximum lateral pulse wave amplitude observed on the left and right walls. Interestingly, the corresponding location of maximum axial pulse wave amplitude after the vessel origin occurs slightly further along the vessel toward the optic disc boundary, that is, after a slight delay. Although the underlying causes of asymmetric collapse remain unknown, this method provides an opportunity to expand our understanding of the phenomenon and explore its relationship to disease and vessel geometry.

The proposed method quantifies sub-pixel vessel wall displacements along retinal vessels within the optic disc, enabling also the characterization of asymmetric lateral vessel wall motion. This method provides an alternative to established approaches for quantifying vessel wall motion such as DVA, PhS-FF-SS-OCT, and modified photoplethysmography. DVA measures retinal vessel diameters in fundus images by analyzing intensity profiles across vessel cross-sections to identify the vessel edges and calculate the distance between them.^7^ However, a previous study reported that DVA cannot quantify lateral pulsation within the optic disc due to the high reflectance of the optic disc in fundus ophthalmoscope images.^10^ PhS-FF-SS-OCT estimates axial retinal motion based on temporal changes in the phase of the OCT signal, and modified photoplethysmography quantifies intensity changes in retinal vessels.^11^^,^^15^ Neither of these two techniques assesses lateral vessel wall motion.

Previous lateral measurement techniques such as DVA measure changes in cross sectional diameter over time.^7^^,^^35^ This implicitly assumes that the vessel axis is unchanging between frames and that wall motion occurs perpendicular to this axis over time. In the case of asymmetric vessel wall pulsation, this is not true. Our method addresses this by non-rigidly warping all frames to a common coordinate space. Then, for each frame, in turn, it measures displacements orthogonal to the vessel centerline in this space based on the inverse warping back to the frame space.

There are several limitations with our method. First and foremost, the primary limitation of the results presented in this paper is the small sample size (3 subjects). Additionally, the three healthy young subjects (aged 26–34) are not representative of the typically older patient populations in which vascular analysis would be applied clinically. The accuracy of vessel wall motion measurement depends directly on the quality of the registration process. A limitation of our existing implementation in MATLAB is that it first globally aligns video frames to the sharpest frame and then performs groupwise non-rigid registration. To avoid the potential bias introduced by initially globally aligning to a single reference frame, another approach would be to perform global groupwise rigid/affine alignment first and then groupwise non-rigid registration. This could be implemented using a toolkit such as ANTs, for example.^36^ Another limitation is that the fidelity of the measurements is constrained by the spatial resolution, frame rate, and noise characteristics of the acquired video dictated by the equipment used. A Canon 5D mark III camera at 25 frames per second was used for this study. Another limitation of this study is that it does not include an assessment of operator dependence with respect to the manual tracing of the vessel of interest on the template image. Lastly, a potential limitation of this method is that the quality of the model fits, assessed in terms of pseudo-*R*^2^, progressively deteriorates away from the optic disc center. This may limit the applicability of this technique beyond the optic disc.

This method enables the continuous sub-pixel quantitative analysis of asymmetric lateral vessel wall motion along the length of retinal vessels within the optic disc using optic disc videos. The detection of asymmetric lateral pulsation in inferior veins within the optic disc across all three healthy young subjects demonstrates the capability of this method to identify spatially localized differences in vessel wall behavior. This provides a foundation for future studies to investigate asymmetric vessel pulsation, its relationship to disease and vessel geometry, and to further advance our understanding of retinal vein pulsation within the optic disc.

## Supplementary Material

Supplement 1
