# Supplementary material for: Quantifying Lateral Pulsation in Retinal Vessels Within the Optic Disc
Source: Transl Vis Sci Technol. 2026 Jul 14;15(7):20. doi: 10.1167/tvst.15.7.20 (PMC13387277; doi:10.1167/tvst.15.7.20)
Supplement: Supplement 1 [file tvst-15-7-20_s001.docx]

Supplementary Material

Pseudocode – Non-Rigid Registration

The groupwise registration process aligns globally registered frames to a mean intensity template computed from the globally aligned stack. This is performed iteratively, with each iteration aligning the frames to a template generated from the previous iteration. Subsequently, pairwise registration maps each locally aligned frame back to its corresponding globally aligned frame.

Step 1. Compute the initial mean intensity template from the globally aligned stack of frames.

Step 2. Deform each globally aligned frame towards the initial mean intensity template (groupwise registration), producing a locally aligned stack and a forward mapping displacement field stack.

Step 3. Repeat Steps 1 and 2 using a new mean intensity template computed from the locally aligned stack. Continue this process until the change in the cost function between successive iterations falls below a predefined threshold value (MATLAB default: $1\times{10}^{-6}$).

Step 4. Compute the reverse mapping displacement field by deforming (pairwise registration) each locally aligned frame obtained in Step 3 to its corresponding frame in the globally aligned stack from Step 1.


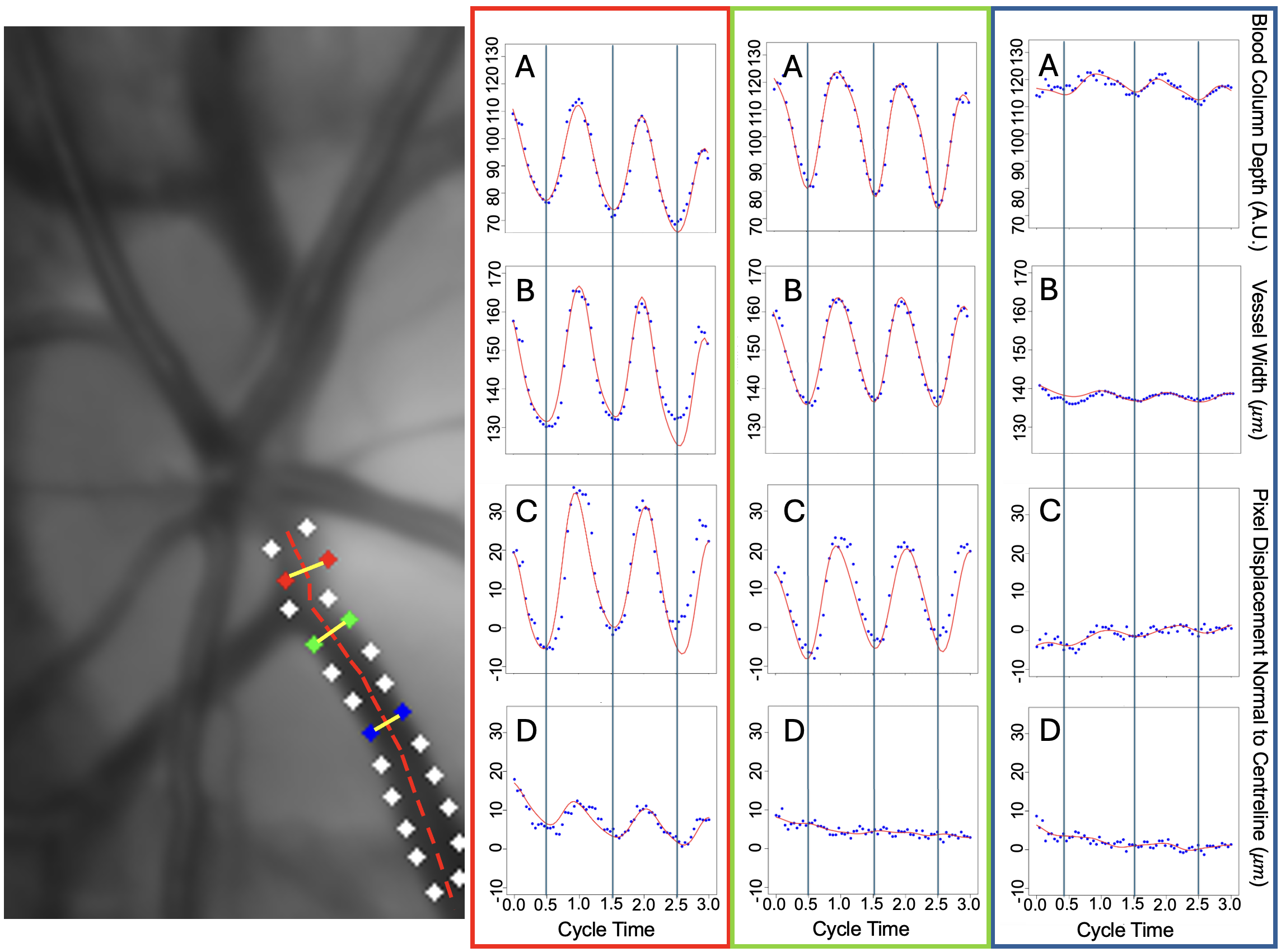


Figure SA1. Harmonic regression model fits for a vertically oriented inferior vein of Subject B over three cardiac cycles. Every tenth edge pixel is marked with a white square. Fits are shown at three yellow crosslines located at the tenth, thirtieth, and sixtieth pixels along the vein centreline (red dashed line) shown in red, green and blue respectively. Blue points are the raw measurements, and the red curve is the fitted model. (A) Axial vessel diameter changes (A.U.). (B) Planar vessel diameter (VD) changes (μm). (C) Right wall (RW) displacements (μm). (D) Left wall displacements (μm).

Table SA1: Summary of pseudo-$R^{2}$ values for the regression models fitted to the planar and axial displacement/diameter measurements along the selected vessel for Subject B. Reported as median (IQR).

| Measurement | Entire length of the vein^*^ | Visibly pulsating segment^†^ |
| --- | --- | --- |
| Right wall (RW) displacement | 0.86 (IQR 0.30) | 0.94 (IQR 0.04) |
| Left wall (LW) displacement | 0.73 (IQR 0.03) | 0.74 (IQR 0.18) |
| Planar vessel diameter (VD) | 0.76 (IQR 0.60) | 0.90 (IQR 0.09) |
| Axial diameter | 0.91 (IQR 0.24) | 0.97 (IQR 0.01) |

* 839 μm; † 455 μm


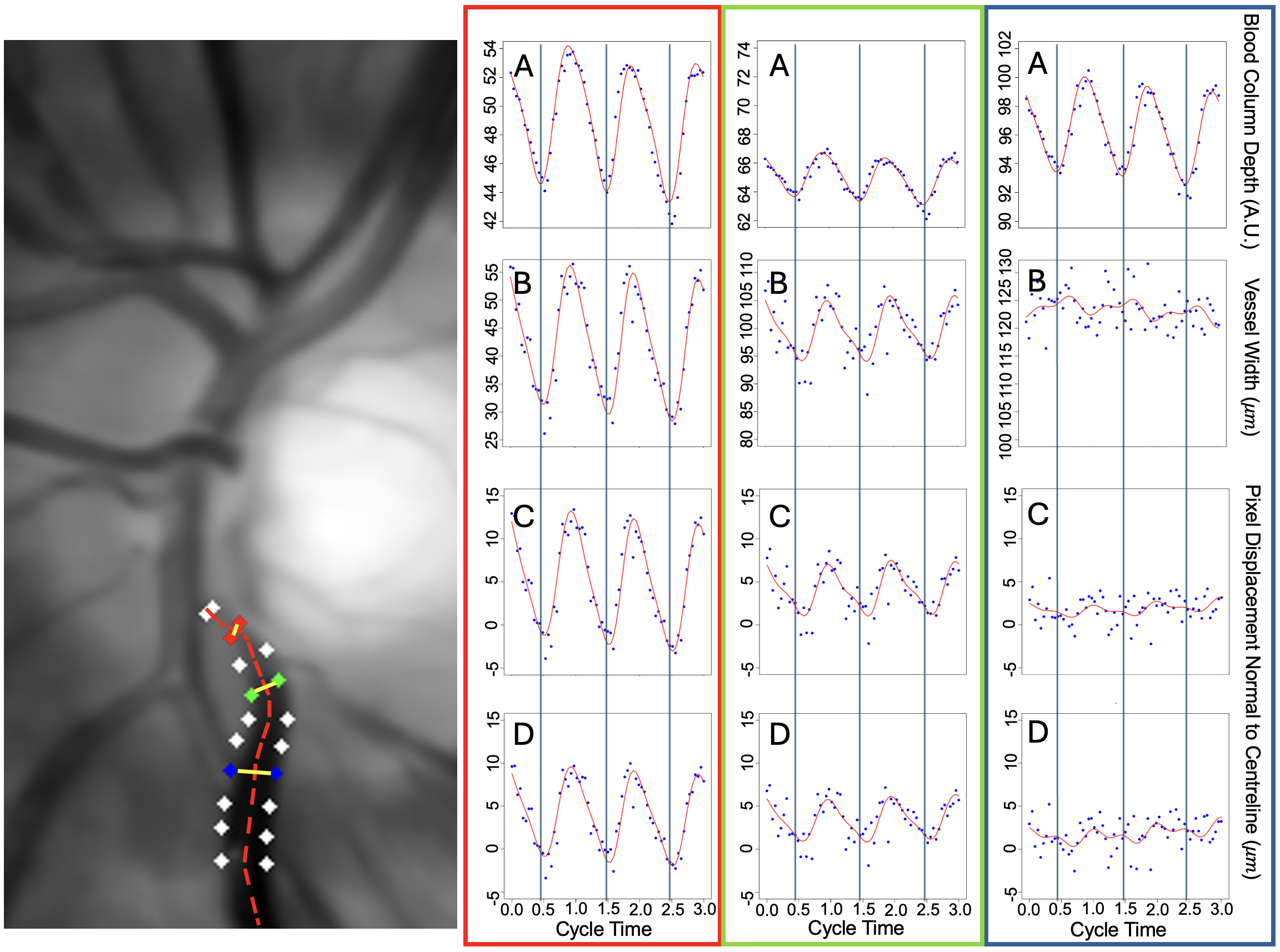


Figure SA2. Harmonic regression model fits for a vertically oriented inferior vein of Subject C over three cardiac cycles. Every tenth edge pixel is marked with a white square. Fits are shown at three yellow crosslines located at the tenth, thirtieth, and sixtieth pixels along the vein centreline (red dashed line) shown in red, green and blue respectively. Blue points are the raw measurements, and the red curve is the fitted model. (A) Axial vessel diameter changes (A.U.). (B) Planar vessel diameter (VD) changes (μm). (C) Right wall (RW) displacements (μm). (D) Left wall displacements (μm).

Table SA2: Summary of pseudo-$R^{2}$ values for the regression models fitted to the planar and axial displacement/diameter measurements along the selected vessel for Subject C. Reported as median (IQR).

| Measurement | Entire length of the vein^*^ | Visibly pulsating segment^†^ |
| --- | --- | --- |
| Right wall (RW) displacement | 0.56 (IQR 0.58) | 0.91 (IQR 0.02) |
| Left wall (LW) displacement | 0.50 (IQR 0.62) | 0.86 (IQR 0.05) |
| Planar vessel diameter (VD) | 0.54 (IQR 0.62) | 0.89 (IQR 0.02) |
| Axial diameter | 0.91 (IQR 0.05) | 0.97 (IQR 0.01) |

* 528 μm; † 189 μm

*
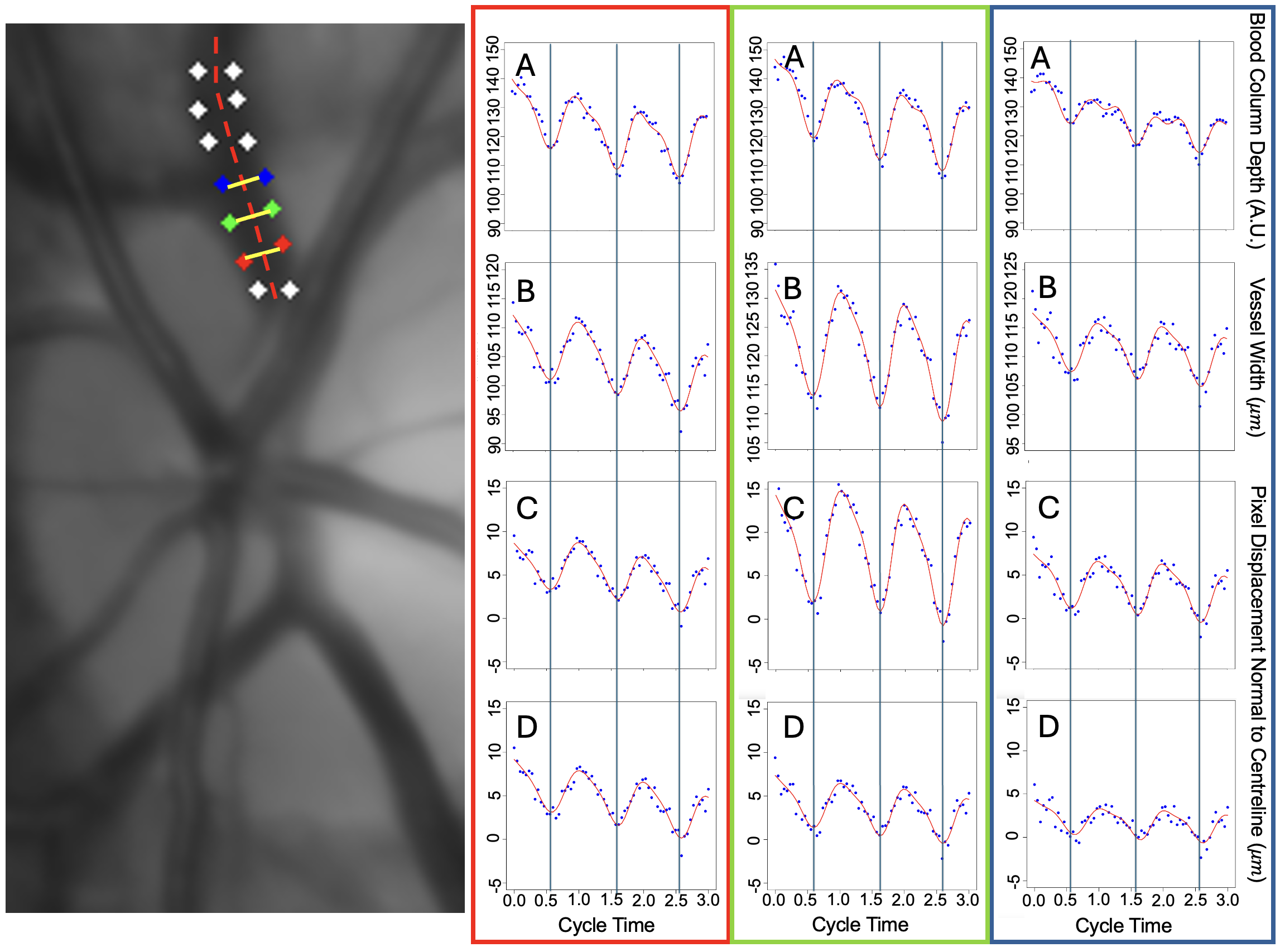
*

Figure SA3. Harmonic regression model fits for a vertically oriented superior vein of Subject A over three cardiac cycles. Every tenth edge pixel is marked with a white square. Fits are shown at three yellow crosslines located at the tenth, twentieth, and thirtieth pixels along the vein centreline (red dashed line) shown in red, green and blue respectively. Blue points are the raw measurements, and the red curve is the fitted model. (A) Axial vessel diameter changes (A.U.). (B) lateral vessel diameter (VD) changes (μm). (C) Right wall (RW) displacements (μm). (D) Left wall displacements (μm).


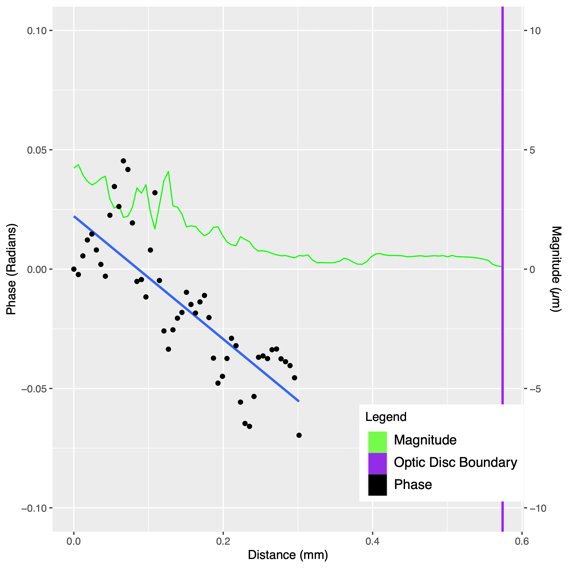

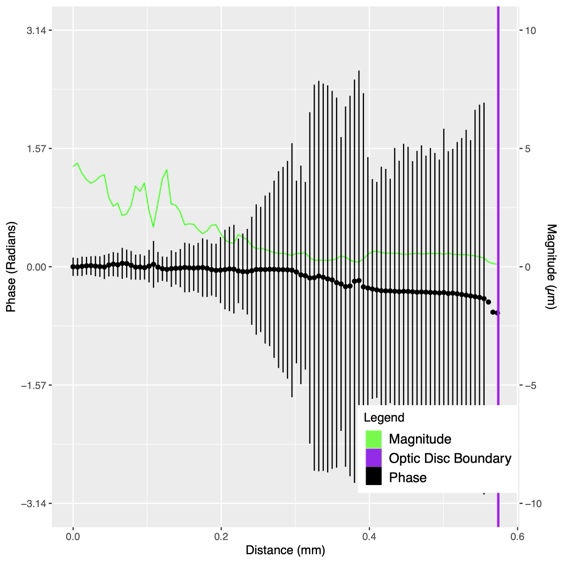


B

A

Figure SA4. Panel A shows plots of phase (black) and magnitude versus distance along the left edge of the inferior vein from the centre of the optic disc for Subject A. The slope of the fitted regression line is used to calculate a pulse wave velocity of 21.14 mm/s. Panel B shows corresponding confidence interval widths for the estimated phase values in Panel A.


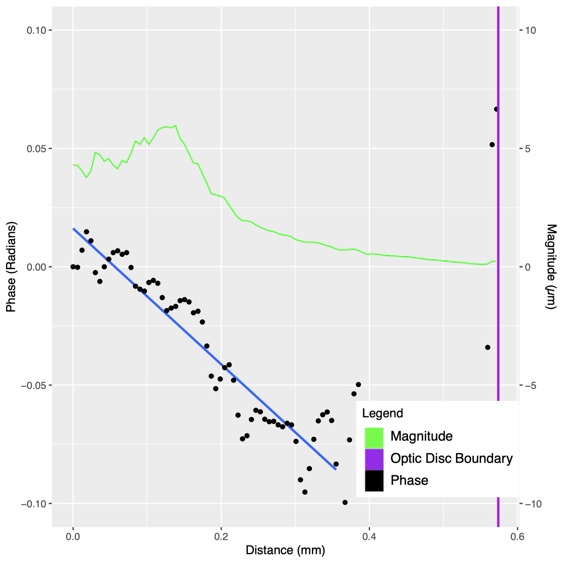

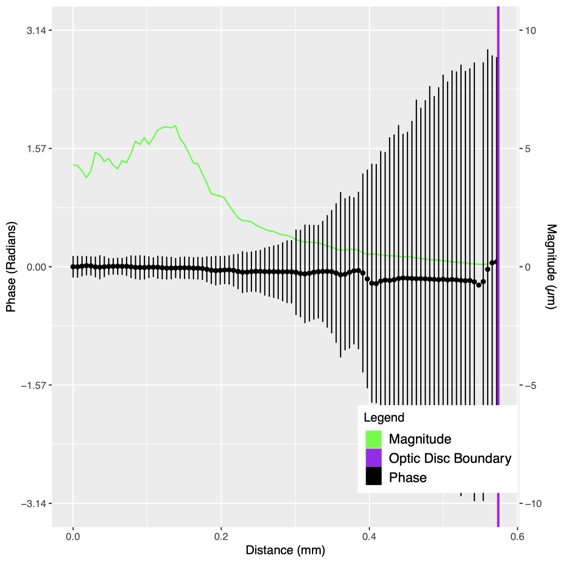


A

B

Figure SA5. Panel A shows plots of phase (black) and magnitude versus distance along the right edge of the inferior vein from the centre of the optic disc for Subject A. The slope of the fitted regression line is used to calculate a pulse wave velocity of 23.38 mm/s. Panel B shows corresponding confidence interval widths for the estimated phase values in Panel A.
